# Personalized prediction of repetitive transcranial magnetic stimulation clinical response in medication-refractory depression data

**DOI:** 10.1016/j.dib.2021.107264

**Published:** 2021-07-14

**Authors:** Helene Hopman, Sandra Chan, Winnie Chu, Hanna Lu, Chun-Yu Tse, Steven Chau, Linda Lam, Arthur Mak, Sebastiaan Neggers

**Affiliations:** a Department of Psychiatry, The Chinese University of Hong Kong, G30, G/F, Multicentre, Tai Po Hospital. 9 Chuen On Road, Tai Po, New Territories, Hong Kong, China; b Department of Radiology, The Chinese University of Hong Kong, Hong Kong, China; c Department of Psychology, The Chinese University of Hong Kong, Hong Kong, China; d Brain Center Rudolf Magnus, University Medical Center Utrecht, Utrecht, the Netherlands; e Department of Social and Behavioural Sciences, City University of Hong Kong, Hong Kong, China

**Keywords:** Depression, Resting-state functional magnetic resonance imaging, Functional connectivity, Neuroimaging, Biomarkers, Transcranial magnetic stimulation, Machine learning, Support vector machine

## Abstract

This article describes a dataset that was generated as part of the article: Personalized prediction of transcranial magnetic stimulation clinical response in patients with treatment-refractory depression using neuroimaging biomarkers and machine learning (DOI: 10.1016/j.jad.2021.04.081). We collected resting-state functional Magnetic Resonance Imaging data from 70 medication-refractory depressed subjects before undergoing four weeks of repetitive transcranial magnetic stimulation targeting the left dorsolateral prefrontal cortex. The data presented here include information about the seed-based analyses such as regions of interest, individual/group functional connectivity maps and contrast maps. The contrast maps are controlled for age, gender, duration of the current depressive episode, duration since the first depressive episode, and symptom scores. Demographics, clinical characteristics, and categorical treatment response variables are reported as well. Further, the individual connectivity values of the identified neuroimaging biomarkers of long-term clinical response were used as features in the support vector machine models are presented in combination with the trained classifiers of the support vector machine models. Post hoc analyses that were not published in the original analyses are presented as well. Finally, the R or MATLAB code scripts for all figures published in the co-submitted paper are included.

## Specifications Table

**Table:** 

Subject	Neuroscience: Biological Psychiatry
Specific subject area	Resting-state functional connectivity in medication-refractory depressed subjects and repetitive transcranial magnetic stimulation treatment clinical response
Type of data	Excel file: Demographics, clinical characteristics and clinical response variables Image: NIfTI files: - Regions of interest used for the seed-based analyses - Individual functional connectivity maps (Fisher transformed Z scores) of the seed-based analyses (seeds: left DLPFC and sgACC) - Average group functional connectivity maps - Group contrast connectivity maps - Short-term responders versus nonresponders - Long-term responders versus nonresponders - Sustained response versus relapse Fig.: 5 .jpg files Table: 5 .docx files Code - Rcode or MATLAB scripts for creating figures
How data were acquired	fMRI scans: Philips 3T MRI scanner Functional connectivity maps/contrasts: CONN toolbox v18b Figures: R version 3.6.1 or MATLAB R2019b
Data format	Analyzed
Parameters for data collection	Resting-state functional magnetic resonance: 6 min - eyes open. Montgomary-Åsberg Depression Rating Scale to measure clinical response. Covariates: age (years), gender (male/female), duration of the current depressive episode (weeks), duration since the first episode (years). Other: age onset, number of depressive episodes, medication, level of treatment refractoriness, education (years)
Description of data collection	Six minutes resting-state functional magnetic resonance imaging data were collected before 20 sessions of repetitive transcranial magnetic stimulation to the left dorsolateral prefrontal cortex. Symptom scores were measured with the Montgomary-Åsberg Depression Rating Scale at baseline, week 4 (end of treatment) and week 12 (2 months post-treatment). Short-term and long-term clinical response were defined as a minimal reduction of 50% in symptom scores at week 4 and 12, respectively. Seed-based analyses were performed with left dorsolateral prefrontal cortex and subgenual anterior cingulate as seeds and controlled for covariates (voxel threshold *p* < .005, cluster threshold *p*-FDR < .05, two-tailed). The support vector machine analyses used the four connectivity markers of long-term clinical response as features. Classifiers were trained on 70% of the data using different feature combinations, and 5-fold cross-validation to minimize overfitting. The trained classifiers were used on the remaining 30% of the dataset to examine generalizability and identify the best model.
Data source location	Institution: The Chinese University of Hong Kong City/Town/Region: Shatin, New Territories Country: Hong Kong SAR China Institution: Prince of Wales Hospital City/Town/Region: Shatin, New Territories Country: Hong Kong SAR China Latitude and longitude (and GPS coordinates, if possible) for collected samples/data: 22.380864578322733, 114.20197324637053
Data accessibility	https://data.mendeley.com/datasets/26dgps7tsg/1 doi: 10.17632/26dgps7tsg.1
Related research article	H.J. Hopman, S.S.M. Chan, W.C.W Chu, H. Lu, C-Y Tsé, S.W.H. Chau, L.C.W. Lam, A.D.P. Mak, S.F.W. Neggers, Personalized prediction of transcranial magnetic stimulation clinical response in patients with treatment-refractory depression using neuroimaging biomarkers and machine learning, *Journal of Affective Disorders, 290,* 261-271. https://doi.org/10.1016/j.jad.2021.04.081

## Value of the Data

• The data provide information about biomarkers of left dorsolateral prefrontal cortex rTMS clinical response in medication-refractory depressed subjects and the impact of two resting-state fMRI denoising strategies, including component based noise correction and global signal regression.
• Researchers may benefit from this data by using the biomarkers and trained support vector machine classifiers as the foundation for a prospective clinical trial to examine the validity of these biomarkers.
• Researchers can benefit from this data for meta-analyses that focus on biomarkers of any antidepressant treatments.

## 1 Data Description

The data are organized in different folders in Mendeley data [1].

*Anticorrelations***:** coordinates_anticorrelation.m script was used to extract the most anticorrelated area in the left middle frontal gyrus and subcallosal cortex using the automated anatomical labeling atlas (AAL) after component based noise correction with and without global signal regression. A 5mm sphere was created around these coordinates. ROI files are saved in anticorrelated_ROI.

*Contrasts_CC***:** All contrast files after pre-processing with component based noise correction (5 NIfTI and 5 text files) of the seed-based analyses. The basename of each file consists of 3 unique parts SBA_’A’_’B’_’C’.nii. A indicates whether the full sample or training sample was used (full or train). B corresponds to the seed region (DLPFC or sgACC). C shows what contrast the file contains (RvsN = Responders > Nonresponders; SvsR = Sustained > Relapse). Each of these files consist of 2 volumes (dim 74 × 92 × 78). Volume 1 shows the statistical T values of the significant clusters and Volume 2 shows the same clusters with discrete values. The row numbers of the MNI coordinates in the text file with the same basename correspond to the discrete values in volume 2.

*Contrasts***:** contrast files after pre-processing with global signal regression (2 NIfTI and 2 text files) comparing long-term responders and nonresponders in the full sample using DLPFC and sgACC as seeds. The naming is similar as in the contrast_CC folder.

*Figures***:** This folder contains all 5 figures in .jpg format.

**Fig. 1:**
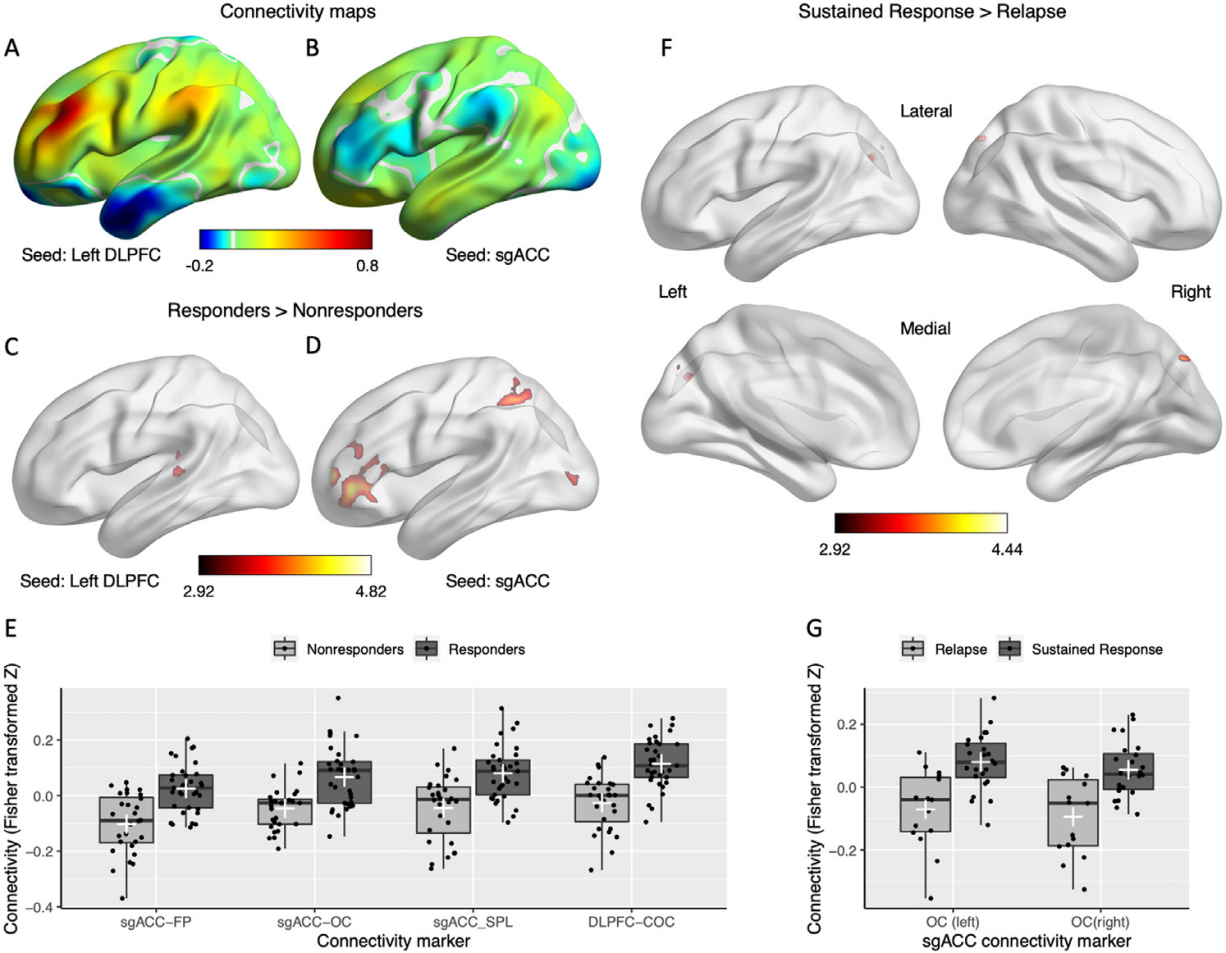
Panel A and B show volume 1 and 2 of the FCmaps_average_CC.nii file, respectively. Panel C, D and F show the contrast files contrasts_CC/SBA_full_DLPFC_RvsN.nii, contrasts_CC/SBA_full_sgACC_RvsN.nii and contrasts_CC/SBA_full_sgACC_SvsR.nii respectively. Brain images were visualized with the BrainNet Viewer (Xia et al., 2013, http://www.nitrc.org/projects/bnv/). Individual connectivity values were extracted and used to create the graphs in panel E and G. Data and code can be found in Plots_code_data.xlxs and Hopman_Rcode.R.

*Hopman_Fig. 1.jpg:* This figure consists of seven (A-G) panels. Panel A and B show volume 1 and 2 of the FCmaps_average_CC.nii file, respectively. Panel C, D and F show the contrast files contrasts_CC/SBA_full_DLPFC_RvsN.nii, contrasts_CC/SBA_full_sgACC_RvsN.nii and contrasts_CC/SBA_full_sgACC_SvsR.nii respectively. Brain images were visualized with the BrainNet Viewer (Xia et al., 2013, http://www.nitrc.org/projects/bnv/). Individual connectivity values were extracted and used to create the graphs in panel E and G. Data and code can be found in Plots_code_data.xlxs and Hopman_Rcode.R

**Fig. 2:**
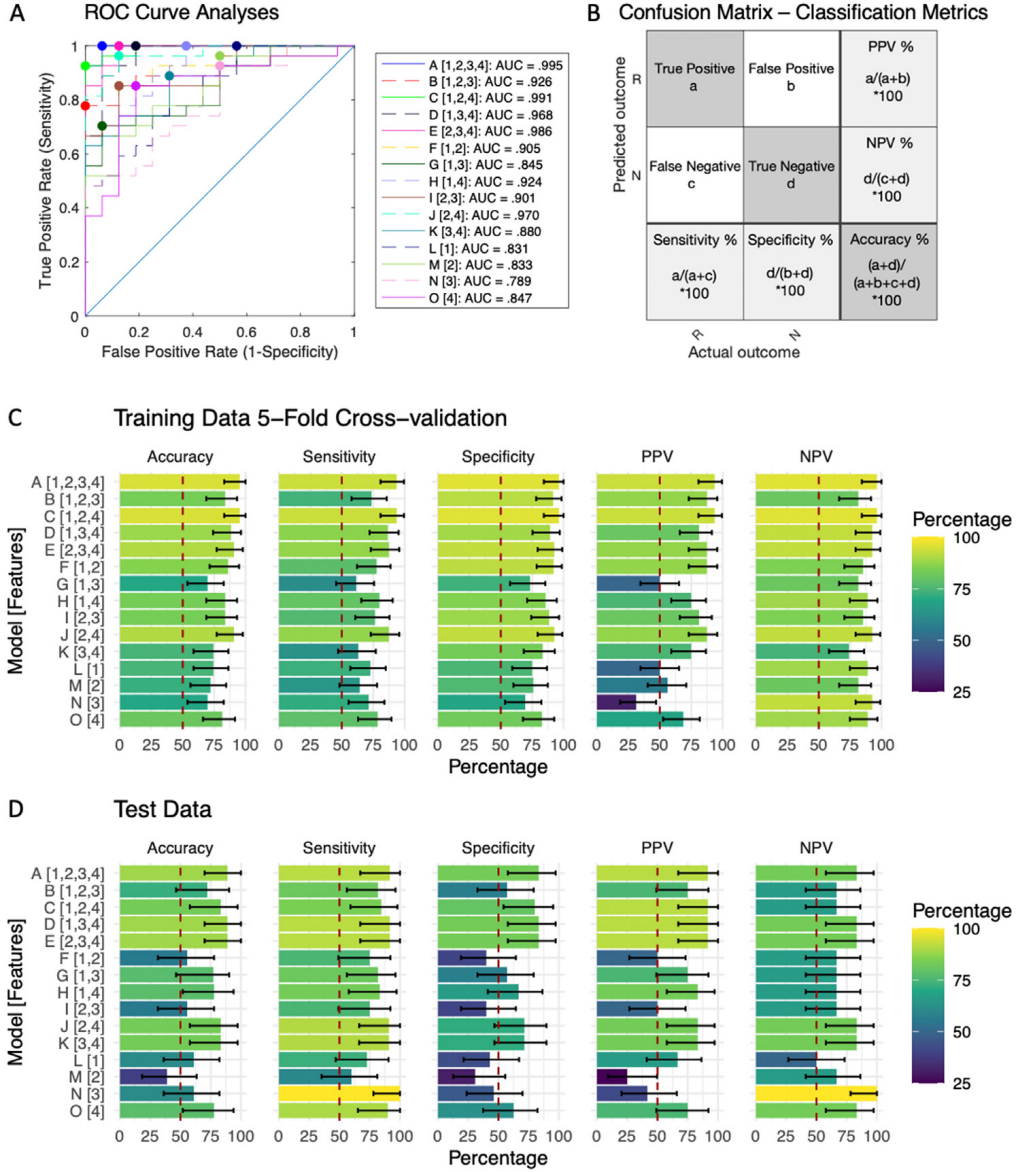
Panel A was created with MATLABR2019b using the files in the /matlab folder. To re-create it in MATLAB, change directory to the matlab folder open and run the ROC_classification_metrics.m script. Panel C and D were based on the output data of the ROC_classification_metrics.m script and variables of interests were manually saved in Hopman_data.xlxs (sheet: SVM) The code for the plots can be found in Hopman_Rcode.R.

*Hopman_Fig. 2.jpg:* This figure consists of 4 panels. Panel A was created with MATLABR2019b using the files in the /matlab folder. To re-create it in MATLAB, change directory to the matlab folder open and run the ROC_classification_metrics.m script. Panel C and D were based on the output data of the ROC_classification_metrics.m script and variables of interests were manually saved in Hopman_data.xlxs (sheet: SVM) The code for the plots can be found in Hopman_Rcode.R.

**Fig. 3:**
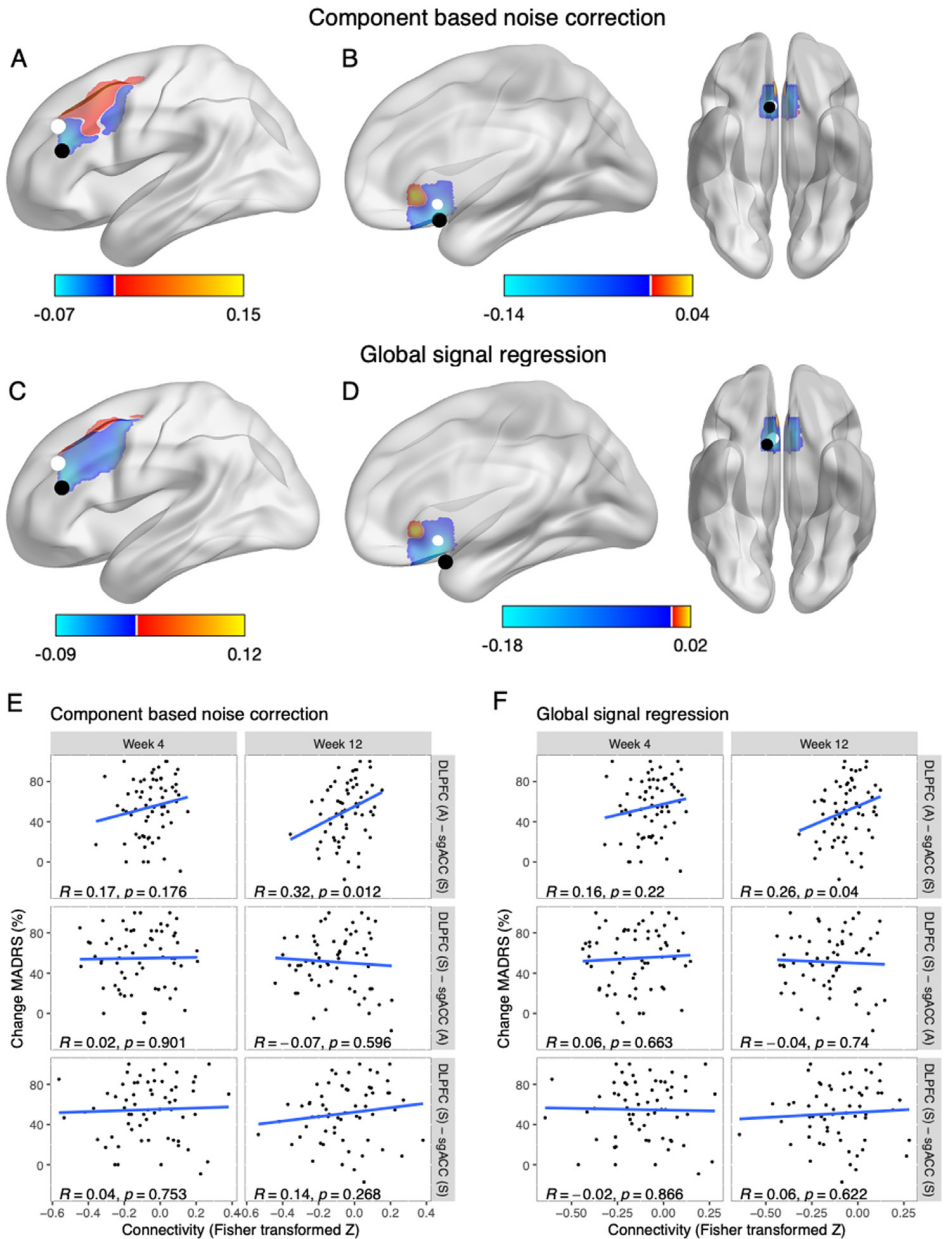
Brain images (Panel A-D) were visualized with BrainNet Viewer (Xia et al., 2013, http://www.nitrc.org/projects/bnv/). Panel A and C show the average functional connectivity map (seed: sgACC) and most anticorrelated spots. The most anticorrelated spots were calculated with the matlab script: coordinates_anticorrelation.m. Then, correlation values were extracted, which can be found in Hopman_data.xlsx (sheet: anticorrelations). This file was also used to create the scatterplots in R, code can be found in Hopman_Rcode.R.

*Hopman_Fig. 3.jpg:* This figure consists of 6 panels. Brain images (Panel A-D) were visualized with the BrainNet Viewer (Xia et al., 2013, http://www.nitrc.org/projects/bnv/). Panel A and C show the average functional connectivity map (seed: sgACC) and most anticorrelated spots. The most anticorrelated spots were calculated with the matlab script: coordinates_anticorrelation.m. Then, correlation values were extracted, which can be found in Hopman_data.xlsx (sheet: anticorrelations). This file was also used to create the scatterplots in R, code can be found in Hopman_Rcode.R.

**Fig. 4:**
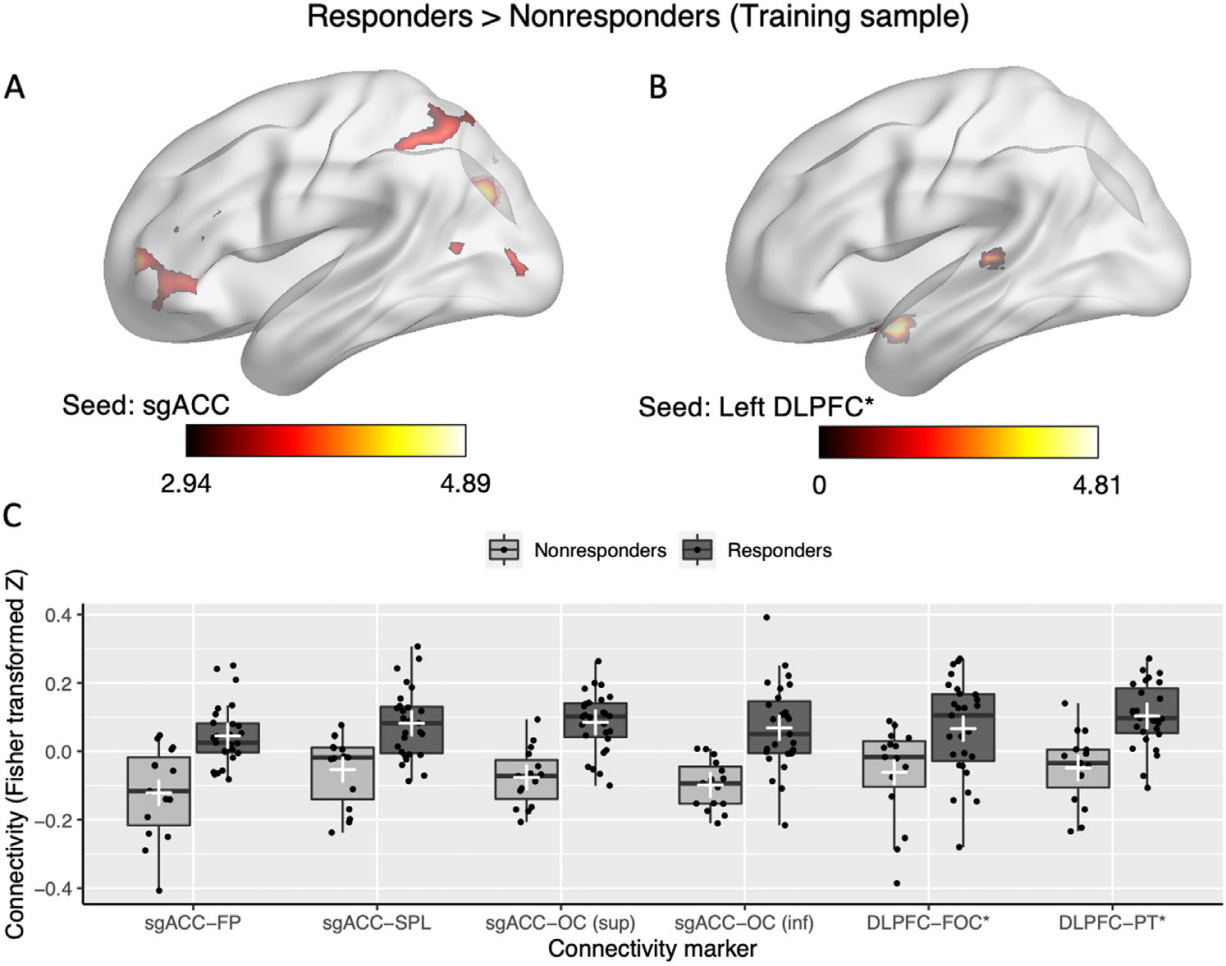
Brain images (Panel A and B) were visualized with BrainNet Viewer (Xia et al., 2013, http://www.nitrc.org/projects/bnv/). Panel A and B show the images /contrast_CC/SBA_train_sgACC_RvsN.nii and /contrast_CC/SBA_train_DLPFC_RvsN.nii, respectively. The connectivity values illustrated in Panel C are saved in Hopman_data.xlsx (sheet: SBA_train_RvsN) and the Rcode can be found in Rcode/Hopman_Rcode.R.

*Hopman_Fig. 4.jpg:* This figure consists of 6 Panels. Brain images (Panel A/B) were visualized with the BrainNet Viewer (Xia et al., 2013, http://www.nitrc.org/projects/bnv/). Panel A and B show the images /contrast_CC/SBA_train_sgACC_RvsN.nii and /contrast_CC/SBA_train_DLPFC_RvsN.nii, respectively. The connectivity values illustrated in Panel C are saved in Hopman_data.xlsx (sheet: SBA_train_RvsN) and the Rcode can be found in Rcode/Hopman_Rcode.R.

**Fig. 5:**
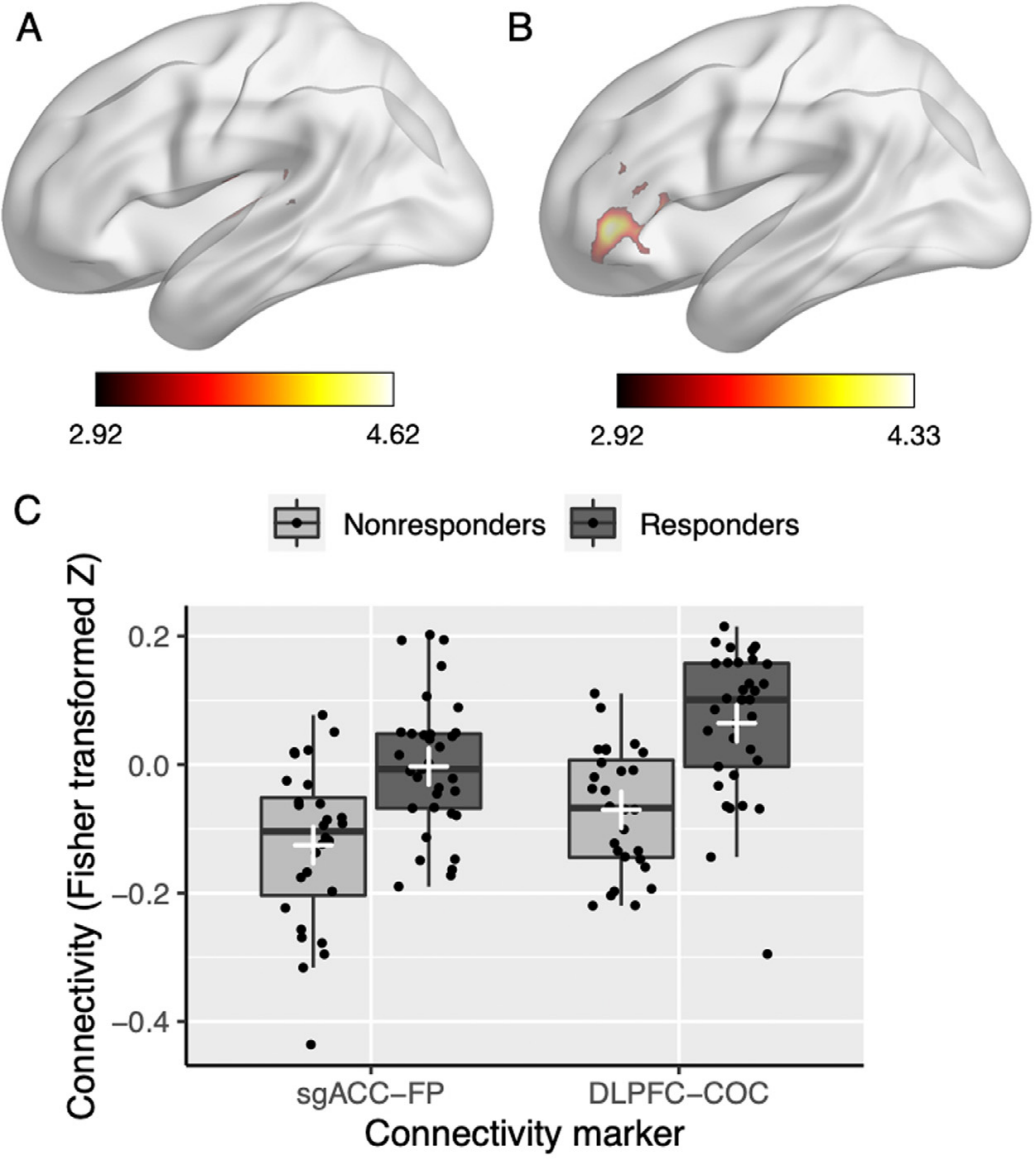
Results of the seed-based analyses after global signal regression. Brain images (Panel A and B) were visualized with BrainNet Viewer (Xia et al., 2013, http://www.nitrc.org/projects/bnv/) using the files in the contrast_GSR folder. The connectivity values illustrated in Panel C are saved in Hopman_data.xlsx (sheet:SBA_RvsN_GSR) and the Rcode can be found in Rcode/Hopman_Rcode.R.

*Hopman_Fig. 5.jpg:* This figure shows the seed-based analyses after global signal regression. Brain images (Panel A/B) were visualized with BrainNet Viewer (Xia et al., 2013, http://www.nitrc.org/projects/bnv/) using the files in the contrast_GSR folder. The connectivity values illustrated in Panel C are saved in Hopman_data.xlsx (sheet:SBA_RvsN_GSR) and the Rcode can be found in Rcode/Hopman_Rcode.R

*Functional_connectivity_maps + ROIs***:** The content of each file is described below. All XX in the filenames is replaced by either CC or GSR, indicating the applied denoising pre-processing strategy.

*FCmaps_average_XX.nii:* average standardized MNI space group level functional connectivity maps of the seed-based analyses (2 volumes; dim 91 × 109 × 91; volume 1 = DLPFC, volume 2 = sgACC). The connectivity maps of subjects with excessive head movement were excluded (SID 2,48,49,58).

*FCmap_DLPFC_individual_XX.nii:* standardized MNI space subject-level functional connectivity maps of the left DLPFC seed-based analysis (67 volumes; dim 91 × 109 × 109). The volume number is similar to the subject ID (SID). The values represent the Fisher transformed Z scores between the mean time series within the left DLPFC seed and that particular voxel.

*FCmap_sgACC_individual_XX.nii:* standardized MNI space subject-level functional connectivity maps of the sgACC seed-to-voxel analysis (67 volumes; dim 91 × 109 × 109). The volume number is similar to the subject ID (SID). The values represent the Fisher transformed Z scores between the mean time series within the sgACC seed and that particular voxel.

*Hopman_SBA_SEED.nii:* MNI space regions of interest used for the seed-based analyses. One volume (dim 91 × 109 × 91) coded with 1 for left DLPFC, and 2 for sgACC.

*Matlab***:** ROC_classification_metrics.m is the script used to perform the ROC analyses and extract classification metrics. Further, this folder contains the trained classifiers of all models (A–O).

*Rcode***:** This folder contains two R scripts. Hopman_Rcode.R was used to create Figs. 1E, 1G, 2C, 2D, 3E, 3F, 4C and 5C. The ANOVA_binomial_logistic_clinical_characteristics.R was used to perform the repeated measures ANOVA and binomial logistic regression reported in Table 1. Both scripts import data from the Hopman_data.xlsx file in the root folder.

**Table 1:** Demographics and clinical characteristics at baseline for all participants (n = 70), short-term (n = 63) and long-term (n = 61) rTMS treatment response and repeated measures ANOVA and binomial logistic regression results.

				Short-term (*n* = 63)		Long-term (*n =* 61)	
		All (*n* = 70)		R (*n* = 41)	N (*n* = 22)	R (*n* = 33)	N (*n* = 28)
Demographics							
Gender, M:F (% Male)		16:54 (29.63)		8:33 (24.24)	8:14 (57.14)	9:24 (37.50)	5:23 (21.74)
Age (years) ± SD		41.93 ± 11.67		45.44 ± 10.38	37.55 ± 11.75	43.76 ± 11.35	40.96 ± 11.62
Education (years) ± SD		12.04 ± 3.18		11.73 ± 2.95	12.50 ± 3.91	11.88 ± 2.78	11.86 ± 3.84
Clinical characteristics							
Age onset (years) ± SD		32.00 ± 11.18	34.44 ± 10.14		28.95 ± 12.32	33.45 ± 11.24	31.21 ± 11.40
Duration ± SD							
Episode (weeks)		28.90 ± 17.19	27.02 ± 16.89		31.18 ± 17.18	26.97 ± 17.10	30.00 ± 16.71
Total (years)		9.93 ± 7.36	11.00 ± 7.68		8.59 ± 6.80	10.30 ± 8.03	9.75 ± 7.00
Episodes (no.) ± SD		3.33 ± 1.71	3.51 ± 1.50		3.00 ± 2.07	3.39 ± 1.95	3.29 ± 1.51
MADRS ± SD		29.67 ± 6.07	29.20 ± 5.50		29.36 ± 7.06	29.85 ± 5.91	29.18 ± 6.00
Medication							
None		4	2		1	2	1
Antidepressant		13	6		4	2	7
Antidepressant and Psychotropic^a^		53	33		17	29	20
TR^b^							
Level 1		9	6		2	3	5
Level 2		34	19		11	15	13
Level 3		26	15		9	14	10
Level 4		1	0		1	1	0

Repeated Measures ANOVA		Time, *β* ± SE	Response,*Β* ± SE		Interaction *β* ± SE	F (3,128)	*p*

Age (years)		2.53 ± 3.08	6.33 ± 2.89*		-3.80 ± 4.04	1.87	0.14
Age onset (years)		1.48 (3.05)	3.79 (2.86)		-2.16 (3.99)	0.70	0.55
Duration (weeks)		-2.08 ± 4.62	-6.50 ± 4.33		2.31 ± 6.05	1.08	0.36
Duration total (years)		1.05 ± 2.00	2.54 ± 1.87		-1.65 ± 2.62	0.69	0.56
Education (years)		-0.51 ± 0.88	-0.56 ± 0.83		0.62 ± 1.15	0.17	0.92
Episodes (no.)		0.28 ± 0.46	0.57 ± 0.43		-0.38 ± 0.60	0.65	0.59
MADRS		-0.06 ± 1.61	0.12 ± 1.51		0.65 ± 2.11	0.12	0.95
Binomial Logistic Regression^c^							

Variable	Gender		Medication			TR^b^	Time model

Time, *β* (SE)	0.59 ± 0.75		-1.10 ± 1.13			-1.61 ± 1.10	-0.49 ± 0.36
Var (dummy 1), *β* (SE)	0.78 ± 0.58		-0.92 ± 1.30			0.54 ± 0.89	-
Var (dummy 2), *β* (SE)	-		-0.46 ± 1.19			-0.63 ± 0.91	-
Time x dummy 1, *β* (SE)	-1.41 ± 0.85+		-0.47 ± 1.82			1.11 ± 1.21	-
Time x dummy 2, *β* (SE)	-		0.82 ± 1.58			1.45 ± 1.23	-
Model summary							
AIC	182.56		183.40			187.86	181.39
Model vs Time Chisq (Δ df)	2.82 (2)		5.99 (4)			1.53 (4)	-

F, Female; M, Male; MADRS, Montgomery-Åsberg Depression Rating Scale; N, nonresponders; R, responders.; TR, treatment refractoriness. * p < .05, + p < .10.

a Psychotropic medication refers to all other classes of medication but antidepressants, including stimulants, antipsychotics, mood stabilizers, and antianxiety agents.

b Treatment refractoriness is based on the criteria by Thase and Rush (1997), a higher level means more treatment resistance.

c For the binomial logistic regression analyses for Gender, Medication and Treatment Refractoriness, respectively, male, no medication, and TR level 1 were used as reference groups. Dummy 1 were female, antidepressants and TR level 2. Dummy 2 were antidepressant + psychotropic and TR level 3 + 4.

*Tables***:** This folder contains 4 .docx file showing the tables.

*Hopman_Table 1.docx:* This table shows the demographics and clinical characteristics for all subjects and by short-term and long-term response. Further, analyses of variance and binomial regression were performed to examine any differences across time points and responder group. All variables are saved in Hopman_data.xlsx (sheet: demo_clinical) and Rcode for these analyses can be found in Rcode/ANOVA_binomial_logistic_clinical characteristics.R.

*Hopman_Table 2.docx***:** This table shows all significant clusters of the seed-based analyses after component based noise correction. The contrasts were long-term responders versus nonresponders and sustained response versus relapse.

*Hopman_Table 3.docx:* Replication of the Seed-Based Analyses using the Training Sample, contrast: long-term responders versus nonresponders.

*Hopman_Table 4.docx:* This table shows all significant clusters of the seed-based analyses after global signal regression. The contrasts were long-term responders versus nonresponders and sustained response versus relapse.

Hopman_data.xlsx: file with all variables used for the analyses. The first sheet (Overview) gives the definition of each variable per sheet. The scripts in the Rcode folder need this file to run.

## 2 Experimental Design, Materials and Methods

### 2.1 Methods

The derived data described in this article are shared at Mendeley Data [1] and were used to support the findings presented in the article: “Personalized prediction of transcranial magnetic stimulation clinical response in patients with treatment-refractory depression using neuroimaging biomarkers and machine learning” [2].

### 2.2 Participants

Participants (*n* = 70) were referred by psychiatrists from the specialist outpatient clinics in the public sector funded by the local government of Hong Kong Special Administrative Region. Participants were right-handed, aged 18–57 years, met the criteria for major depressive disorder (MDD) based on the Diagnostic and Statistical Manual of Mental Disorders (DSM-IV; principle axis I diagnosis), moderate or severe episode defined by a scored of ≥ 20 on the Montgomery-Åsberg Depression Rating Scale (MADRS; [3]) and had failed to respond adequately to at least one full course (>6 weeks) of antidepressant medication or were medication intolerant (Table 1). Participants were screened to exclude confounding factors such as significant head trauma, active abuse of alcohol or illegal substances, other DSM-IV axis II disorders, and neurological disorders. Participants were also excluded if they had received other neuromodulation treatments in the preceding year. For safety reasons, participants that reported suicide ideation or recent suicide attempts, or with a contraindication for the use of functional Magnetic Resonance Imaging (fMRI, e.g. pacemakers, metal implants, pregnancy; for details refer to [4]) and repetitive Transcranial Magnetic Stimulation (rTMS, e.g. history of seizures/epilepsy; for details refer to [5] were also excluded). Finally, participants with psychotic symptoms were excluded, because research showed poor response rates in this group [6]. Each participant provided written informed consent and received a travel cost compensation.

### 2.3 Measures

Several demographic and clinical variables were collected during the pre-treatment measurement, including age, gender, handedness, years of education, duration of the current depressive episode in weeks, total duration since the first depressive episode, the number of depressive episodes, medication, and the level of treatment refractoriness [7]. Clinical assessments were administered by a research psychiatrist. The first assessment was before the pre-treatment brain scan and consisted of the Structured Clinical Interview for DSM-IV to ascertain current and lifetime Axis I and II psychiatric diagnoses [8]. The MADRS [3], Hamilton Depression Rating Scale [9], Clinical Global Impression scale [10], and Global Assessment of Functioning score [11] were also administered. Further, the Chinese version of the Beck Depression Inventory-II [12] was completed by the participants. The baseline symptom score measures were validated before the first rTMS session and reassessed at week 2, 4, 6, 8, and 12 using the MADRS [3], Hamilton Depression Rating Scale [9], Clinical Global Impression Scale [10], and Beck Depression Inventory-II [12]. In this paper, the MADRS is used as the primary outcome measure. This clinician-administered scale has high inter-rater reliability and was designed to be sensitive to antidepressant treatment effects in patients with MDD [13]. The MADRS consists of ten items that are rated on a 0–6 continuum (0 = no abnormality, 6 = severe). A last observation carried forward approach was applied for participants with a missing week 12 outcome variable using the week 6 (*n* = 1) or week 8 measurement (*n* = 1) instead. The percentage change in MADRS symptom scores at week 4 (MADRS_baseline_ - MADRS_wk4_)/MADRS_baseline_ * 100%) and week 12 (MADRS_baseline_ - MADRS_wk12_)/MADRS_baseline_ * 100%) were calculated. Response was defined as a minimum reduction of 50% in symptom score measured with the MADRS [3] immediately after the last treatment and two months post-treatment compared to baseline. These two time points will be further referred to as short-term and long-term categorical treatment response. Responders were divided into two groups for additional analyses to examine pre-treatment connectivity associated with relapse. The relapse group refers to participants that only showed short-term categorical treatment response. The sustained response group refers to patients that showed both short-term and long-term categorical treatment response.

### 2.4 Brain scans

#### 2.4.1 Image acquisition

MRI scans were acquired up to two weeks before the start of the rTMS treatment on a 3.0T Philips Achieva Medical Scanner with an eight-channel SENSE head coil (Philips Healthcare, The Netherlands) at the Prince of Wales Hospital, Hong Kong Special Administrative Region in China. The first scan was a high resolution T1-weighted structural scan covering the whole brain acquired with the following parameters: repetition time = 7.54 ms, echo time = 3.53 ms, flip angle = 8⁰, 1.1 × 1.1 × 0.6 mm voxels, number of slices = 285, slice orientation = sagittal, slice thickness = 1.2 mm, Field of View = 250 mm^3^, and matrix size = 240 × 240. This scan was used to register with the resting-state fMRI data, and for segmentation into grey matter, white matter, and cerebrospinal fluid, and normalization to template space. The T1-structural scan was followed by a six-minute resting-state fMRI scan consisting of 170 volumes with the following parameters: repetition time = 2050ms, echo time = 25ms, flip angle = 90⁰, 3.2 mm^3^ voxels, slice thickness = 3.2 mm, Field of View = 205 mm², and matrix size = 64 × 64. Research has shown that six minutes of resting-state fMRI results in moderate to strong reliability for functional connectivity measures [14], [27]. Further, a T2-weighted scan and diffusion-weighted imaging scan were collected, but these scans are beyond the scope of this paper. The total scan duration was 25 min and 20 s.

#### 2.4.2 Image pre-processing

Resting-state fMRI data were pre-processed using the default pipeline of the CONN toolbox v18.b [15]. The pre-processing steps included realignment and unwarping, temporal slice time correction, functional outlier detection (ART-based identification of outlier scans), segmentation, normalization, and smoothing (8 mm Gaussian kernel). Two denoising strategies were separately examined including component based noise correction with and without global signal regression [15], [16]. For component based noise correction, the following parameters were regressed out: white matter (10 dimensions), cerebrospinal fluid (5 dimensions), realignment parameters (6 dimensions), scrubbing (61 dimensions), and the effect of pre (1 dimension). For global signal regression, the same procedure was performed, but additionally, a brain mask of the entire brain was added as a region of interest to measure the average brain signal, which was also added as a regressor (1 dimension). Subsequently, a default temporal band-pass filter (.008–09 Hz) and detrending were applied for both pre-processing methods.

The region of interests for the seed-based analyses were defined in MNI standardized space. For the left DLPFC, a 20-mm sphere was drawn around previously determined optimum stimulation Montreal Neurological Institute (MNI coordinates: X = -46, Y = 45, Z = 38 [17], [18]. The sphere was masked by a cortical brain mask to exclude voxels outside the brain. The size of this ROI was based on previous research that showed that figure-of-eight shaped coils stimulated neurons in a cortical area of 2–3 cm^2^ and to a depth of approximately 2 cm [19]. For sgACC, the same method described by Fox and colleagues [17] was used, i.e. a 10-mm sphere was drawn around the MNI coordinates 6, 16, -10, and masked by a cortical brain mask to exclude subcortical voxels.

### 2.5 Repetitive transcranial magnetic stimulation protocol

Participants received 20 sessions of neuronavigated rTMS to the left DLPFC over 4 weeks. Individual sessions consisted of 30 min of 10 Hz rTMS (3000 pulses; 30-s cycles, 5 s on, 25 s off). A Magstim Super-Rapid device was used with a 70-mm figure-of-eight double air film coil (Magstim Ltd, UK) and manually centred at MNI coordinates X = -46, Y = 45, Z = 38 (Talairach X = -45, Y = 45, Z = 35; [18]) using Brainsight TMS neuronavigation (Rogue Resolutions Ltd, UK). The resting motor threshold was defined as the minimum TMS intensity that elicited a motor-evoked potential of ≥ 50μV peak to peak in the contralateral abductor pollicis brevis in 5 out of 10 trials. The motor threshold was measured before the first treatment and after 10 sessions. The stimulation output was 120% of the motor threshold. The stimulation output was adjusted to 100% for three participants that could not tolerate 120%. The post hoc analysis showed that the inclusion or exclusion of these participants did not influence the results.

### 2.6 Statistical analyses

#### 2.6.1 Demographics and clinical characteristics

Repeated measures analysis of variance (ANOVA) and binomial logistic regression were performed to examine differences in demographics and clinical characteristics. For the continuous variables, repeated measures ANOVA were performed with two main terms including Time (2 levels: short-term, long-term) and Response (2 levels: responder, nonresponder) and one interaction term (Time x Response). For the categorical variables, binomial logistic regression analyses were performed with Response (0 or 1) as outcome variable. Time and each categorical variable were added as predictors and dummy coded. For time (2 levels: short-term, long-term), short-term was the reference group. For gender (2 levels: male, female), the male group was the reference group. For medication (3 levels: none, antidepressants, antidepressant + psychotropic), the none group was the reference group, antidepressants was dummy 1 and antidepressant + psychotropic was dummy 2. For treatment refractoriness (3 levels: 1, 2, 3+4), level 1 was the reference group, level 2 was dummy 1 and level 3+4 was dummy 2. Model comparison was performed using Akaike Information Criteria (smaller is better criterion) and Chi-square test (Table 1).

#### 2.6.2 Seed-based analyses

Subject-level bivariate Pearson's correlations between the mean time series within each seed (DLPFC/sgACC) and the blood-oxygen-level-dependent time series of each voxel in the brain were extracted and converted to normally distributed Fisher transformed z-scores to conform to the assumptions of generalized linear models using the CONN toolbox [15] with short-term and long-term treatment response as the outcome variable, respectively. Analyses were controlled for age, gender, MADRS symptom score at baseline, the duration of the current depressive episode, and total duration since the first depressive episode. The resulting individual seed maps for each region of interest (left DLPFC and sgACC) were used for the second-level analyses comparing responders versus nonresponders at both time points (Figs. 1A–E and 5A–C). Further, seed-based analyses were performed to examine the pre-treatment connectivity differences between patients that showed sustained treatment response and patients that relapsed after the end of the treatment (Fig. 1F and G). The whole-brain results for all seed-based analyses were thresholded twice [20]; voxel-level threshold p < .005, cluster threshold p-FDR < .05, two-sided. The effect-sizes correspond to the beta values of the group variable of each ANCOVA and represent the connectivity difference between groups controlled for all covariates (Tables 2 and 4).

**Table 2:** Significant clusters of the seed-based analyses with left DLPFC and SgACC as Seeds. The contrasts were long-term rTMS responders (n = 33) versus nonresponders (n = 28) and sustained response (n = 24) versus relapse (n = 15) (Voxel Threshold p < .005, cluster threshold p-FDR < .05, Two-tailed).

ID	Coordinates	T_min_	Size	*p* -	Side	Area (no. of voxels)	Effect
No.	MNI x y z		Voxels	FDR			Size
**Responders > Nonresponders**							

Seed: sgACC							

1	-34 +56 +06	2.92	1433	<.001	Left	Frontal pole (814) Inferior frontal gyrus, pars triangularis (272) Middle frontal gyrus (104) Frontal orbital cortex (103) Inferior frontal gyrus, pars opercularis (51) Frontal operculum cortex (16)	.29
2	-30 -76 +08		497	.005	Left	Lateral occipital cortex, inferior (160) Occipital pole (19) Lateral occipital cortex, superior (6)	.26
3	-32 -54 +54		434	.007	Left	Superior parietal lobule (301) Lateral occipital cortex, superior (92) Postcentral gyrus (15)	.30

Seed: DLPFC							

4	-66 -30 +04	2.92	659	.004	Left	Central opercular cortex (135) Planum temporale (134) Superior temporal gyrus, posterior (114) Heschl's gyrus (98) Middle temporal gyrus, posterior (43) Parietal operculum cortex (34)	.30

**Sustained response > Relapse**							

Seed: sgACC							

	-28 +76 +12	2.92	409	.024	Left	Lateral occipital cortex, superior (134) Cuneal Cortex (59) Precuneous Cortex (31)	.15
	+26 -76 +16		370	.024	Right	Lateral occipital cortex, superior (151) Lateral occipital cortex, inferior (33) Cuneal Cortex (12) Precuneous Cortex (9)	.16

Seed: DLPFC							

*No significant clusters (voxel threshold p <.005, cluster threshold p-FDR < .05)*							

DLPFC, dorsolateral prefrontal cortex; FDR, false discovery rate; MNI, Montreal Neurological Institute coordinates; sgACC, subgenual anterior cingulate cortex

**Table 3:** Replication of the seed-based analyses using the training sample.

ID	Coordinates	T_min_	Size	*p* -	Side	Area (no. of voxels)	Effect
No.	MNI x y z		Voxels	FDR			Size
**Seed: sgACC**							

1	-34 -56 +06	2.94	605	0.003	Left	Frontal pole (458) Inferior frontal gyrus, pars triangularis (92) Frontal orbital cortex (22)	.33
2	-32 -62 +54		571	0.003	Left	Superior parietal lobule (295) Lateral occipital cortex, superior (225) Postcentral gyrus (24)	.30
3	-28 -72 +30		456	0.008	Left	Lateral occipital cortex, superior (252) Middle temporal gyrus, temporooccipital (52) Lateral occipital cortex, inferior (179)	.29
4	-34 -76 +06		323	0.030	Left	Lateral occipital cortex, inferior (17) Occipital pole (5)	.29

Seed: DLPFC				***p*-uncorr**			

*No significant results with voxel threshold p < 0.005, cluster threshold p-FDR < .05.*							

1.	-26 +06 -18	2.94	158	0.026	Left	Frontal Orbital Cortex (51) Amygdala (16) Temporal pole (11) Insular cortex (9)	.29
2	-50 -28 +04		118	0.0498	Left	Planum temporale (40) Superior temporal gyrus, posterior (36) Middle temporal gyrus, posterior (8)	.31

**Table 4:** Significant clusters of the seed-based analyses with left DLPFC and SgACC as seeds after global signal regression. the contrasts were long-term rTMS responders (n = 33) versus nonresponders (n = 28) and sustained response (n = 24) versus relapse (n = 15) (voxel threshold p < .005, cluster threshold p-FDR < .05, two-tailed).

ID	Coordinates	T_min_	Size	*p* -	Side	Area (no. of voxels)	Effect
No.	MNI x y z		Voxels	FDR			Size
**Responders > Nonresponders**							

Seed: sgACC							

1	-46 +42 -02	2.92	652	.003	Left	Frontal pole (343) Inferior frontal gyrus, pars triangularis (176) Frontal orbital cortex (54) Inferior frontal gyrus, pars opercularis (29) Frontal operculum cortex (6) Middle frontal gyrus (5)	.29

Seed: DLPFC							

4	-48 -28 +04	2.92	529	.012	Left	Central opercular cortex (152) Heschl's gyrus (113) Planum temporale (101) Superior temporal gyrus, posterior (53) Middle temporal gyrus, posterior (26) Parietal operculum cortex (19)	.29

**Sustained response > Relapse**							

Seed: sgACC							

*No significant clusters (voxel threshold p <.005, cluster threshold p-FDR < .05)*							

Seed: DLPFC							
*No significant clusters (voxel threshold p <.005, cluster threshold p-FDR < .05)*							

DLPFC, dorsolateral prefrontal cortex; FDR, false discovery rate; MNI, Montreal Neurological Institute coordinates; sgACC, subgenual anterior cingulate cortex

#### 2.6.3 Supervised machine learning

Machine learning was applied to examine whether combining the identified biomarkers could increase the accuracy of categorical rTMS treatment response prediction. Our sample was split into a training/validation dataset (70%) and a test dataset (30%). We used MATLAB's Machine Learning toolbox (The Mathworks, Natick, MA) to search for the best classification model type, including decision trees, discriminant analysis, support vector machines, logistic regression, nearest neighbors, naive Bayes, and ensemble classification. Hyperparameters optimization was automated by the toolbox. Subject-level Fisher transformed z-scores identified by the seed-based analyses above were entered as features, and long-term categorical treatment response was entered as a binary outcome (responders/nonresponders). The average accuracy scores and prediction speed from the 5-fold cross-validation procedures were used to determine the best classification model type. This classification model type was used to train classifiers for all feature combinations in the training/CV dataset. The trained classifiers subsequently were used to examine performance in the independent test dataset. A large decrease in performance in the test dataset compared to the training/CV dataset suggests overfitting [21]. The SVM classifiers returned the validationPredictions and validationScores for the training data, which were used to create confusion matrices and perform ROC curve analyses, respectively. The ROC curve analyses were performed with MATLAB's perfcurve function. This function returned the optimal operating point of the ROC curve as an array of size 1-by-2 with False Positive Rate and True Positive Rate values for the optimal ROC operating point. The optimal operating point was obtained by finding the slope, S, using S = (cost(P|N)-cost(N|N))/(cost(N|P)-cost(P|P)) * N/P where cost(I|J) is the cost of assigning an observation of class J to class I, and P=True Positive + False Negative and N=True Negative + False Positive are the total observation counts in the positive and negative class, respectively. This function subsequently identified the optimal operating point by moving the straight line with slope S from the upper left corner of the ROC plot (False Positive Rate=0, True Positive Rate=1) down and to the right until intersecting the ROC curve (Fig. 2A). According to the literature, AUC is an effective summarize measure of the diagnostic ability of a test. An AUC between 0.7 and 0.8 is considered acceptable, between 0.8 and 0.9 is considered excellent, and higher than 0.9 is considered outstanding [22]. Confusion matrices were used to calculate classification metrics for the optimal operating points of each model (Fig. 2B), including sensitivity, specificity, positive predictive value, negative predictive value, and accuracy (Fig. 2C and D). Sensitivity is the proportion of responders that is correctly classified, while specificity is the proportion of nonresponders that is correctly classified [23], [24]. The positive and negative predictive values reflect the probability that participants with a positive or negative test truly become responders or nonresponders, respectively [18], [19]. The 95% binominal confidence intervals (CI) were calculated to determine statistical significance [25].

### 2.7 Post hoc analyses

Based on the reviewers’ comments, two post hoc analyses were performed which were not included in the original article.

#### 2.7.1 Correlational analyses anticorrelation

Correlational analyses were performed to directly examine the predictive value of the most anticorrelated areas. Functional connectivity maps were masked with the automated anatomical labeling atlas (AAL) regions of interest, including left middle frontal gyrus and subcallosal cortex (Fig. 3A–D). Then, the voxel coordinates of the lowest connectivity value were extracted within each masked area. The fslmaths function (FMRIB Software Library v6.0.2) was used to draw a 5mm kernel around the most anticorrelated coordinates resulting in two new regions of interest; DLPFC (A) and sgACC (A). Connectivity values between the DLPFC and sgACC seeds and new anticorrelated regions of interest were extracted and correlational analyses were performed to examine its relationship with rTMS treatment response (Fig. 3E and F).

#### 2.7.2 Seed-based analyses training data

In the original article [2], validity of the results were questioned because the selected features for machine learning were based on the functional connectivity group differences of the full sample. It was suggested by one of the reviewers to examine whether the seed-based analyses results would still hold by using the training dataset only. Therefore, we performed additional second-level analysis comparing long-term responders and nonresponders using the training dataset. Analyses were controlled for age, gender, MADRS symptom score at baseline, the duration of the current depressive episode, and total duration since the first depressive episode. As the DLPFC seed analysis did not reveal any significant clusters, it was examined whether this was the result of the decreased power due to a smaller sample size by using a more liberal cluster threshold (cluster-size p-uncorrected < .05 instead of p-FDR < 0.05; Fig. 4, Table 3).

## Ethics Statement

This study was approved by the Joint Chinese University of Hong Kong–New Territories East Cluster Clinical Research Ethics Committee [Ref No.: CRE-2014.041] and in line with the Helsinki Declaration [26]. The study was also registered at ClinicalTrials.gov [ID: NCT03348761].

## CRediT Author Statement

**Helene Hopman:** Conceptualization, Data Curation, Formal Analysis, Investigation, Methodology, Visualization, Writing - original draft, Writing - review & editing; **Sandra Chan:** Conceptualization, Funding acquisition, Investigation, Methodology, Project administration, Supervision, Writing - review & editing; **Winnie Chu:** Conceptualization, Methodology; **Hanna Lu:** Writing - review & editing; **Chun-Yu Tse**: Writing - review & editing; **Steven Chau:** Writing - review & editing; **Linda Lam:** Conceptualization, Methodology; **Arthur Mak:** Conceptualization, Methodology; **Sebastiaan Neggers:** Conceptualization, Methodology, Supervision, Writing - review & editing.

## Declaration of Competing Interest

S. Neggers holds a minority share in Brain Science Tools BV, a company manufacturing stereotactic navigation technology for TMS. This did not influence the design, analysis, or reporting of the current manuscript in any way. No potential conflict of interest was reported by the other authors.
